# Healthcare worker’s views and experiences of factors impacting confidence to provide perinatal bereavement support: a qualitative study in Malawi and Zimbabwe

**DOI:** 10.1080/16549716.2026.2709907

**Published:** 2026-08-03

**Authors:** Idesi Chilinda, Unice Goshomi, Debbie M. Smith, Angela Chimwaza, Tina Lavender, Tracey A. Mills

**Affiliations:** a Adult Health Nursing Department, Kamuzu University of Health Sciences, Blantyre, Malawi; b Research Innovation and Postgraduate Centre, Women’s University in Africa, Harare, Zimbabwe; c Division of Psychology and Mental Health, The University of Manchester, Manchester, UK; d Department of International Public Health, Liverpool School of Tropical Medicine, Liverpool, UK

**Keywords:** Qualitative, stillbirth, bereavement support, healthcare workers, confidence

## Abstract

**Background:**

Worldwide, around 1.9 million babies are stillborn, and 2.5 million newborns die soon after birth annually, the overwhelming majority occur in low- and middle-income countries (LMICs). Perinatal death has devastating impacts for families and communities, but not all parents receive adequate respectful bereavement support. Healthcare workers in LMICs often report that bereavement support is challenging, but few studies have explored factors that impact their confidence to deliver optimal care.

**Objective:**

To explore healthcare worker’s views and experiences of factors impacting their confidence to provide bereavement support to parents after stillbirth or neonatal death in Malawi and Zimbabwe.

**Methods:**

Using qualitative approaches, a purposive sample of healthcare workers in four maternity facilities in Malawi and Zimbabwe was recruited. Guided by the principles of information power, six dual-moderated focus groups supplemented with eight individual in-depth interviews were conducted. The focus group discussions and interviews were audio-recorded and transcribed verbatim. Data were analyzed using the framework method.

**Results:**

Sixty-six healthcare workers, including midwives, nurses, service managers, nurse educators, and doctors, participated. Two main themes were identified: (1) ‘*at least we tried our best’* reflecting individual factors including feelings of inadequacy and insufficient education, and (2) ‘*as a facility we are not quite there yet’* characterized by a lack of organizational support, feeling unsupported and a lack of continuing professional development.

**Conclusion:**

Findings highlight the need to address the individual and environmental barriers to providing support to bereaved women and families. Improving access to pre- and in-service education and training, mentorship and management support are potential solutions.

## Paper Context

- **Main Findings:** Healthcare workers in LMICs lack confidence to provide support to women and families following stillbirths or neonatal deaths.
- **Added knowledge:** Sub-optimal working environments with lack of explicit clinical guidance, unavailability of education and lack of management priority and support are an important influence on lack of confidence.
- **Global health impact for policy and action:** Interventions targeting the enhancement of healthcare worker education and skills through context-specific guidelines and training provide a basis for increasing healthcare worker confidence.

## Background

Around 1.9 million babies are stillborn, and 2.5 million newborns die soon after birth each year, the overwhelming majority in low and middle-income countries (LMICs) [1,2]. Sub-Saharan Africa and South Asia account for more than 75% of global perinatal deaths [2,3]. Each death is a profound tragedy for parents and has far-reaching adverse social and economic consequences, impacting communities and wider society [4]. Studies demonstrate that sensitive and compassionate support from healthcare workers in the period immediately after the death of a baby is crucial in supporting grief and adjustment for parents and families and reducing negative health and social outcomes [4,5]. However, across global settings, including sub-Saharan Africa, many parents do not receive respectful care and support after stillbirth or neonatal death, including during the immediate postnatal period in facilities [5–8]. Insensitive communication from staff, lack of information and limited opportunities for parenting, such as seeing and holding the baby after birth, are reported [7–9]. Poor care contributes to disenfranchised grief. Where the death of the baby is not socially validated, social stigma and isolation particularly impact women [6–9]. Improving perinatal bereavement support has been identified as a priority for global action in maternal and newborn care [10,11].

Healthcare workers in LMIC maternity settings have acknowledged that providing bereavement support is among the most challenging aspects of their role and often lack confidence in this aspect of care [12–14]. Confidence, defined in this context, as the perception of having knowledge and skills to provide bereavement support, is recognized as a key attribute underpinning quality care [12]. Studies in high‑income countries suggest that a combination of factors including access to continuing education, support to develop practice skills and robust organisational endorsement is crucial influences [15]. Studies of healthcare workers’ experiences in caring for parents after stillbirth or neonatal death in sub‑Saharan Africa suggest some transferability of these issues [1,8]. However, contextual and cultural diversity impacting bereavement care in LMIC settings supports the need to better understanding of factors impacting healthcare workers in sub-Saharan Africa. The current study is part of a larger mixed-methods study, and findings will inform the development and validation of a context‑specific tool to assess healthcare workers’ clinical confidence in providing perinatal bereavement care in LMICs. Studies from high‑income countries have informed the conduct of this study due to the paucity of reported evidence from LMICs on health worker clinical confidence in providing perinatal support. The aim of this study was to explore healthcare workers’ views and experiences of factors influencing their confidence to provide perinatal bereavement care in Malawi and Zimbabwe.

## Methods

### Design

This study, part of a wider project, adopted a qualitative descriptive approach. Healthcare workers were recruited from four urban public maternity facilities, in central Malawi (2) and north-eastern Zimbabwe (2) which provide maternity and neonatal care at no cost to service users. Community Engagement and Involvement (CEI) and stakeholder groups, including parents with experience of the death of a baby, clinicians and policy makers, supported the research in both countries from study design to interpretation of the data. This included reviewing interview topic guides to ensure key areas of interest were covered. Following data analysis, the main themes were presented to the stakeholders and CEI groups for discussion and confirmation of the congruence of description. The research team comprised of nurse-midwife educators (Malawi, Zimbabwe, UK), who all had experience of providing perinatal bereavement care and a health psychologist with research experience of bereavement care. To minimize the influence of own assumptions on the research process, researchers maintained awareness of their influences and assumptions during data collection, analysis and interpretation.

### Ethics

Ethical approval was obtained from the Liverpool School of Tropical Medicine Research Ethics Committee (23–006), UK, Kamuzu University of Health Sciences Research and Ethics Committee (COMREC P.05/23/4099), Malawi, Medical Research Council of Zimbabwe and Research Council of Zimbabwe (MRCZ/A/3083). This research was conducted in accordance with the code of ethics (Declaration of Helsinki) for research involving humans.

### Participants and recruitment

A purposive sample of healthcare workers (n = 66) including nurse-midwives and doctors who regularly provided care to bereaved women and families after stillbirth or neonatal death, and managers with responsibility for postnatal or neonatal care services was recruited. Eligible participants had at least one-year of post-registration experience. Healthcare workers in the included facilities were informed about the research during workshops conducted by the research team at each facility and by posters in staff areas. Those interested in participating in focus group discussions (planned 2–4 per country, 8–12 participants per group) contacted the research team directly for further information. This planned sample size was guided by the principles of information power [16].

### Data collection

Dual-moderated focus group discussions were conducted in a private room at the participants’ workplace by experienced moderators with a midwifery/nursing background, in English, which is the predominant language of clinical practice in both countries. Where a convenient time could not be arranged to attend a focus group, an individual interview was offered at a venue of participant’s choice. No-one was present during the interviews except the researchers and the participants. A topic guide, developed based on existing literature and reviewed by the CEI group, bereaved parents and stakeholders, was used. The researchers had prior training in good clinical practice, qualitative research including data collection, analysis and interpretation during their doctoral training and prior to commencement of data collection. Participants neither had prior relationship with the researchers nor knowledge of the interviewers. Data collection was conducted between September 2023 and February 2024 by two female nurse-midwife postdoctoral researchers (IC and UG) who had extensive qualitative research experience. A study-specific questionnaire was used to collect brief demographic and practice data (job title, main area of work, qualifications, duration of practice) prior to the discussions. A participant-led approach was adopted, starting with broad open-ended questions inviting sharing of views and experiences for example ‘*What do you think influences the care you and colleagues give to women and families after the death of a newborn baby in this hospital?’* In FGDs, moderators promoted discussion by encouraging all participants to contribute. Prompts such as *‘can you tell me more about … .’ or ‘what makes you say …* .’ were used to clarify responses and enhance depth when required. No repeat interviews were conducted. A verbal summary was provided to the participants by the moderator/interviewer at the end of the discussions and interviews to confirm the main points of discussion. Interviews were audio-recorded following consent and transcribed verbatim. Participant identity was concealed by using study identification numbers. Contemporaneous field notes were completed following each FGD or interview and reflexive diaries maintained throughout the data collection period.

### Data analysis

Transcripts were analyzed manually using the Framework approach [15]. This systematic, yet flexible method comprised five stages; the first 2 stages were conducted independently by two researchers in each country. Each transcript was read and re-read, line by line for data familiarization. Following this, concepts and initial themes were grouped together to generate the draft coding frameworks. In stage 3, two UK-based researchers (TM and DS) reviewed the draft frameworks and applied these to the whole data set, with input from the Malawi and Zimbabwe researchers, revising the subthemes and themes, which were presented in charts and further refined for coherence in stage 4. Subsequent discussions involving the whole research team were held to agree the final themes, capturing an understanding of factors impacting healthcare workers to provide perinatal bereavement care. The final interpretation was presented to the CEI and stakeholder representatives in each country for confirmation to enhance rigor.

## Results

Six dual-moderated focus groups (2 in Malawi and 4 in Zimbabwe) were conducted with healthcare workers, including registered nurse-midwives (*n* = 35), nurse-midwife technicians (*n* = 11), nurse-educators (*n* = 6), community midwifery assistants (*n* = 3) and a senior nursing officer (*n* = 1). To promote open discussion in Malawi, one FGD included junior midwives (nurse midwife technicians and community midwifery assistants), while senior midwives and senior nursing officers attended a separate group. In Zimbabwe separate FGDs were held with managers and educators and 8 individual in-depth interviews were conducted to accommodate nurse-midwives (*n* = 6), doctors (*n* = 2) who wished to participate but could not attend the scheduled FGDs. None of the participants dropped out of the study. Participants had 1–16 years of clinical maternity experience (Table 1), most worked in labor and postnatal wards, with the remainder practicing in the neonatal nursery, and as educators. FGDs lasted between 45 min and 1 h, while interviews lasted between 30 min and 1 h.

**Table 1.** Participant characteristics.

PARTICIPANT CHARACTERISTICS			TOTAL
	MALAWI	ZIMBABWE	
Participant number	(*n* = 20)	(*n* = 46)	66
Age range	26–42 years	34–56 years	
Median age	32 years	37 years	
**Job title**			
Registered Nurse Midwives	6	37	43
Nurse Midwife Technician	11	0	11
Community Midwifery Assistants	3	0	3
Doctors	0	3	3
Educators	0	6	6
**Main area of work**			
Labour, postnatal &HDU	17	37	54
Neonatal nursery	1	3	4
Antenatal	2	0	2
Education	0	6	6
**Time working in maternity and NNU**			
1–5 years	1O	14	24
6–10 years	8	14	22
more than 10 years	2	18	20
**Highest educational qualification**			
Certificate/Diploma	14	37	51
Degree	6	9	15

Two main themes, with six sub-themes, were identified from the FGDs and interviews which described healthcare workers’ views and experiences on factors influencing confidence. These were common across both countries and all professional groups (Table 2). The theme titles are direct quotes from participants. The first theme ‘*at least we tried our best’* describes individual factors impacting health workers experiences of providing bereavement care, while the second theme ‘*as a facility we are not quite there yet’* focuses on environmental and organizational issues.

**Table 2.** Summary of key themes and sub-themes.

Key themes	Sub-themes
At least we tried our best	Feeling uncertain and inadequate
	Insufficient education
As a facility we are not quite there yet	Lack of organisational support
	Unsupported and unsafe
	Lack of continuing professional development

### Theme 1 – ‘at least we tried our best’

#### Feeling uncertain and inadequate

All participants articulated the importance of healthcare workers providing compassionate support to women and families after the death of a baby. They recognized that a variety of professional competencies, including strong interpersonal skills, were required to build trusting relationships, demonstrate compassion and communicate understandable information:

We need counselling skills so that we will be able to counsel the mothers when they lose their babies … .by counselling skills I mean communicating to the mother and maybe explaining to her the causes of death, neonatal death so that she will understand and accept the outcome. (Labour ward Midwife Zimbabwe)

Many healthcare workers described situations when they felt uncomfortable or incapable in interactions with bereaved women or their relatives. This included being unable to provide satisfactory or acceptable answers to questions. Fearing embarrassment and humiliation in front of patients and colleagues led some heath workers to give incorrect or incomplete information, which they recognized as suboptimal care.

I too do not feel confident to provide care to bereaved families …. she was asking me questions which were beyond my capability so I feel I have the gap in this aspect of care. This gap causes low self-esteem in me and you end up giving false information to the woman. (Registered Nurse Midwife, postnatal ward, Malawi)

In practice, many expressed feeling inadequate in their contacts with grieving women and families and suggested difficulties in ‘getting it right’. Healthcare workers struggled with communication; knowing when to start conversations, how to respond to women most appropriately, what words to use, and ensuring appropriate information was given were frequent worries.

I do not trust myself considering the setting of our facility, is it the right time to counsel the woman, or should I wait for the woman to grieve first and then I should come in? This confuses me. Should I wait for the woman to cry for so long? (Nurse-Midwife Technician, Malawi)

Deficits in knowledge and understanding were compounded by personal emotions. Healthcare workers experienced profound sadness in response to frequent poor outcomes encountered in their settings. They sometimes felt that their ability to openly communicate and offer support to women was hampered by concerns about being blamed for poor outcomes. The perception of ineptitude in difficult situations sometimes became overwhelming and led a few healthcare workers to withdraw entirely, to avoid contact with distressed women and families.

You would feel useless and incompetent to assist the woman who is grieving, you end up feeling useless and even end up breaking down into tears in front of the patient and avoid her seeing it I quickly left the place and went home. (Ward Manager, Maternity Zimbabwe)

#### Insufficient education

In both Malawi and Zimbabwe, healthcare workers felt that their pre-service education did not prepare them adequately for providing skills for perinatal bereavement support. While some key competencies, including communication and counselling, were addressed in their training, the content was rarely specific to bereavement care. Participants felt that their lack of understanding of normal grief responses and parent’s needs diminished their ability to provide sensitive and compassionate care in the immediate period after baby death:

I have partial knowledge of psychological counselling which I did during pre-service, but it’s not that deep that I could competently reach out to support a woman who has lost her baby. (Nurse Midwife Technician, labour ward, Malawi)

Some healthcare workers were able to compensate for a lack of formal skills development by drawing on their own or family experiences of death and loss. Several described how their personal experiences of perinatal death increased their confidence in meeting parent’s needs. For example, recognizing the importance of providing honest explanations to grieving women and giving them sufficient time to process this information.

She didn’t want to accept that the baby is dead. So, I had to explain further and also explain the possible causes and it took my time to explain to her because I once went through that situation. (Midwife, labour ward Zimbabwe)

### Theme 2 – ‘as a facility, we are not quite there yet’

Although their commitment to supporting mothers whose babies had died was clear, healthcare workers in both countries identified significant shortcomings in the services and resources available.

I feel as a facility, we are not yet there to provide bereavement care, as I said the care we provide is fragmented, there is no continuity of care …. (Registered Nurse Midwife, Labour ward, MW)

#### Lack of organizational support

Many healthcare workers acknowledged that the working environment in facilities did not support the effective provision of quality bereavement support. In both countries, facilities did not provide separate rooms for postnatal women whose babies had died. Caring for bereaved women in the same areas as those with live babies limited privacy and inhibited communication with grieving women and their families, increasing trauma.

… we need to separate those bereaving clients from those who have their live babies, they are just mixed with those with their babies and I think they could be provided a room where they are nursed alone unlike them with those with babies. (Postnatal Ward Senior Midwife Zimbabwe)

Healthcare workers also identified a lack of guidance for caring for women after stillbirth or neonatal death in their facilities. Lack of explicit standards and frameworks was perceived as a barrier to consistent quality bereavement support provision. The absence of written guidelines was also interpreted as indicating a lack of priority to bereavement support as an important dimension of care in the organisation.

Since I started working here I have never seen any guidelines or policies to guide our practice. We use the knowledge gained in pre-service and also from our intuition. (Nurse manager, labour ward, Malawi)

I feel that there is need for those guidelines and protocols on how we can be managing these women, and also when they deliver the IUD (intrauterine death) how can we handle them. (Postnatal Ward Midwife Zimbabwe)

Lack of staff and time available to provide perinatal bereavement support were also major issues. Most participants described overwhelming workload, particularly in labor wards, resulting in a lack of priority being given to providing psychological and emotional support to women.

For me to have time for these bereaving mothers there should be staff … we have forty deliveries per day so imagine of the forty, if there is one bereaved mother there is no time to care for her when we are looking to deliver the other forty babies so we really need some. (Labour Ward Midwife Zimbabwe)

#### Unsupported and unsafe

Unsupportive organizational cultures and poor leadership were also identified as having negative impacts on practice. Healthcare workers often described emotional and challenging situations in their contacts with bereaved families. Parents and relatives were understandably distressed and expressed anger on some occasions. Several healthcare workers shared experiences of themselves or colleagues feeling directly blamed for a baby’s death, with complaints related to poor practice, withholding pain relief, and failure to complete basic assessments or interventions.

I once met a woman who blamed me for the loss of her baby, she was very emotional and she was saying ‘aa sister my baby is dead now and you didn’t check my BP (blood pressure) you didn’t give me some analgesia so it means you want to kill me as well’ … ee I was very emotional about it. (Labour Ward Midwife Zimbabwe)

A few health workers related experiences of staff being intimidated or threatened by relatives. One doctor was accused of criminal behavior, with subsequent involvement of law enforcement following a stillbirth.

I was once asked to surrender myself to the police she and her husband does so since the mother claimed that her baby cried and I had exchanged her live one with a dead baby. She claimed that there was no indication that her baby was going to die if so, why was she not told in preparations. (Obstetrician, Zimbabwe)

In these situations, health workers who lacked the skills to respond in complex emotional situations felt further compromised by inadequate support within their workplace. There were several accounts describing managers’ actions as counterproductive, for example openly criticizing individual health workers after poor outcomes without investigating causes. Participants also shared experiences of managers or senior staff directly questioning practice in the presence of women and families which they perceived as fueling complaints and conflict.

You are also accused of negligence by your boss during the handover take over round in front of the patient making them angrier as their suspicion is being confirmed through our conversion in front of the patient and other colleagues. (Labour Ward Midwife Zimbabwe)

Health workers felt that these behaviors encouraged the perception of individual blame for poor outcomes and undermined staff morale. They also acted as a barrier to open communication which is essential for effective bereavement support.

#### Lack of continuing professional development

Access to continuous professional development (CPD) offered a potential strategy for addressing the identified shortcoming pre-service education. However, participants consistently highlighted unavailability of relevant bereavement support training in their facilities.

I have never had any CPD training, sometimes we learn from what our colleagues are doing in practice, then you copy from them, so there was no official training. (Nurse Midwife technician, Postnatal ward, Malawi)

Healthcare workers acknowledged the gaps in their knowledge including normal grief processes, understanding parents’ experiences after death of a baby and the principles of good care during the immediate postnatal period.

I need to develop listening skills and how to communicate to families that are grieving; things such as what information to give and at what point to give the information. (Registered Nurse Midwife, Malawi)

They felt strongly that more formal education was essential to develop skills for practice and improve confidence to provide perinatal bereavement support.

I think for me there is need to be taught or educated about child loss and bereavement care and be taught what exactly would be going on in the individual who lost the baby and what support they need … … .so that they would be able to understand the process maybe they would allow this mother to cry and grieve about their loss. (Labour Ward Midwife Zimbabwe)

The absence of ongoing support to signpost women and families after discharge was also highlighted as an issue. Healthcare workers expressed discomfort with being unable to offer follow-up support that women and families could access particularly to meet ongoing needs for emotional and psychological support which could be lacking in the community.

So maybe we should have some referral points or places, referral centers where they go and get continuous, whether church or counselors too where they can be referred after the care at the hospital. [Maternity Manager Zimbabwe]

## Discussion

This study was the first to explore healthcare workers’ views and experiences of factors influencing their confidence to provide perinatal bereavement support in Malawi and Zimbabwe. Healthcare workers clearly appreciated the importance of providing appropriate support to bereaved mothers and families but personal feelings of inadequacy and uncertainty, caused in part by insufficient knowledge and skills, reduced confidence. Similar concerns have been acknowledged by clinicians in LMICs where structural barriers including a lack of training in bereavement communication, resource constraints, workload and culture impeded efforts to provide perinatal bereavement support [17]. Also, studies conducted in high‑burden countries assert that grieving families perceived the care from health workers as inadequate, limited and not providing opportunities for discussion after the death of the baby [8,9]. This was compounded by cultural and societal stigma in sub-Saharan Africa where stillbirths and newborn deaths are associated with witchcraft and a consequence of parents’ immoral behavior [9,10]. Women describe being blamed for the loss, with limited opportunities to grieve and pressure to conceive again [9]. Further, in some communities, a baby who dies before, during or soon after birth is perceived as ‘less human’, considered unworthy of mourning accorded to other deaths, resulting in rapid burial with secrecy [18].

Sub-optimal working environments were also an important influence with a lack of explicit clinical guidance, unavailability of education and a lack of management priority and support. The current findings confirm previous evidence that health workers in LMICs are profoundly impacted by stillbirth and neonatal death, with emotional impacts similar across settings [19–21]. These challenges were compounded by resource limitations, including a lack of staff and health system deficiencies. Similar findings were reported in a recent study of health workers in Kenya and Uganda [22]. Therefore, it is unsurprising that few healthcare workers in this study expressed security in their knowledge and understanding to provide high‑quality bereavement support, a finding common with other studies in sub-Saharan Africa [18].

In healthcare settings, bereaved women and families expect providers to be physically and emotionally present and offer ongoing support after the death of a baby [22]. Healthcare workers in this study felt unprepared, with perceived lack of communication skills a common issue. Similar concerns have previously been acknowledged across global settings with healthcare workers often expressing concerns around knowing how to initiate conversations and build relationships [22–26]. Deficits in communication skills negatively impacted healthcare workers, who sometimes avoided interactions with bereaved parents in response to feelings of inadequacy [27] potentially further compromising care. Similar to reports from other LMICs, healthcare workers in our study gained skills mainly through informal means observing peers and personal experience [28,29]. However, this can be emotionally draining for peers and self, an experience which may raise internal feelings of failure and questioning one’s competency [30]. Access to adequate bereavement support is an important contributor to improving respectful maternal and newborn which emphasizes rights to dignified and health care [31]. Improvements in curricula and ongoing mentorship to better equip students and staff with the skills to offer perinatal bereavement support and handle emotionally demanding situations are critical [28]. Such strategies may also improve patient outcomes, ensure staff confidence to provide perinatal bereavement support, and increase job satisfaction [26].

The absence of guidelines, pathways and associated development of knowledge and skills for perinatal bereavement support was also identified as a recurrent issue. This finding affirms other studies which emphasizes that absent or inadequate formal guidance for perinatal bereavement support can result in inconsistency or poor quality care by healthcare workers [10,20,26,27]. In these circumstances, practice may be led by intuition, personal beliefs and routine rather than evidence [31], as seen in this study. Other research recommends that culturally appropriate guidelines may positively impact on the confidence and competence of healthcare workers to offer bereavement support and improve health outcomes of bereaved mothers and families [27,29,31–33]. Continuous professional development (CPD) training among healthcare workers, specifically focused on bereavement support, could also support the development of relevant skills and improve levels of confidence. Some high-income countries have bereavement care pathways and guidance including training for health workers to provide compassionate care to parents experiencing perinatal loss [34]. However, strategies and interventions to improve health worker capability to provide bereavement care in LMICs, including Malawi and Zimbabwe, should be based on context appropriate evidence including understanding cultural considerations [35].

Sub-optimal working environments including physical limitation, workloads, and culture impacted healthcare workers’ confidence to provide perinatal bereavement support to mothers and families. A recent systematic review conducted in LMICs suggested that provision of separate spaces to ensure the privacy of bereaved families was important [22,27]. Staff shortages and competing tasks are an issue in high‑income settings [36–38] but affect the capacity of trained healthcare workers more acutely in low-income countries [32]. Our research suggests that these issues persist and need to be addressed to enable healthcare workers to respond to the needs of bereaved women and families. Finally, participants in both countries highlighted a lack of organizational priority impacting on the confidence of attending healthcare workers. Negative practice cultures evidenced in this study have also been reported in Kenya and Uganda [35]. Health workers faced open criticism and abuse from managers following poor perinatal outcomes leading to a lack of open dialogue and learning [35]. Health workers were unprepared and lacked support in responding to parents’ grief including distress and anger, sometimes misinterpreting emotions as personal criticism [39]. These studies demonstrate the importance of supportive leadership, institutional commitment and policy in addition to personal behavior in improving experiences of women and families [35,38].

Future research could benefit from the application of a behavior change model such as the Theoretical Domains Framework (TDF) [40]. Using the TDF in future research will help to identify barriers and enablers to behaviors which can then be the focus of interventions to change behavior. For example, a lack of communication skills was identified as a barrier to bereavement support in the current study. ‘Skills’ is one of the 14 domains in the TDF and thus could help to identify evidence-based strategies to overcome this barrier.

### Strengths and limitations

This study represents the first extensive exploration of healthcare workers’ experiences in sub-Saharan Africa providing an in-depth understanding of complexities impacting confidence in providing perinatal bereavement support. We conducted both in-depth interviews and FGDs, enabling triangulation of the data and enhanced rigor of the findings. Also, we identified significant commonalities in healthcare worker experiences despite differences in samples across the two countries, allowing others to assess transferability of findings to similar settings. We recruited healthcare workers directly involved in providing bereavement support for mothers and families from several facilities in two countries in sub-Saharan Africa with a high burden of stillbirths. The insights gained reflect the gaps of healthcare workers and may guide future evidence-based strategies to improve healthcare confidence. The study had some limitations. FGDs and in-depth interviews were conducted by research midwives with own assumptions, beliefs and prior experience of providing support to women and families with a stillbirth. To minimize the influence of beliefs and assumptions, researchers maintained reflexive accounts of own assumptions after each interview through notetaking and considered during the analysis and interpretation of data, increasing trustworthiness of the study findings. Moreover, different expertise from the research team (Nurses, Midwives and Psychologist) helped to question each other’s interpretation of the data, enhancing trustworthiness of the study findings.

## Conclusion

Deficits in knowledge and skills were key influences on confidence to provide perinatal bereavement support in facilities in Malawi and Zimbabwe. However, health workers also highlighted the critical contribution of organizational and environmental factors to influencing their capability to provide high quality care. Sustained improvement in respectful and compassionate support for parents and families in these and similar settings will require better education for health workers, but also prioritization of bereavement care within organizations and the wider health system. In addition to training, availability of context appropriate evidence-based guidance, provision of spaces to ensure privacy for parents and adequate support and security for health workers are key.

## Supplementary Material

COREQ_Checklistfor submission.pdf

## Data Availability

Data to support findings of this study are available upon request.
